# Effects of Permissive Hypercapnia During Lung Surgery on Inflammatory Biomarkers in Serum and Bronchoalveolar Lavage Fluid: A Randomized Controlled Trial

**DOI:** 10.3390/metabo16080550

**Published:** 2026-08-04

**Authors:** Milena Stojanović, Milena Vasilijic, Jelena Zivadinovic, Milica Randjelovic, Aleksandar Nikolić, Tatjana Jevtovic Stoimenov, Radmilo Janković

**Affiliations:** 1 Clinic for Anaesthesia and Intensive Therapy, University Clinical Center Niš, 18000 Niš, Serbia; mvasilijic4@gmail.com (M.V.); jelena5491@gmail.com (J.Z.); mirandjelovic91@gmail.com (M.R.); dracanikolic@gmail.com (A.N.); jankovic.radmilo@gmail.com (R.J.); 2 School of Medicine, University of Nis, 18000 Niš, Serbia; tatjana.jevtovic.stoimenov@medfak.ni.ac.rs; 3 Department for Biochemistry, School of Medicine, University of Nis, 18000 Niš, Serbia

**Keywords:** permissive hypercapnia, one-lung ventilation, bronchoalveolar lavage fluid, inflammation

## Abstract

Introduction: Permissive hypercapnia has become an integral component of lung-protective ventilation strategies during one-lung ventilation (OLV), particularly in thoracic surgery. Its potential immunomodulatory effects have attracted increasing interest; however, clinical evidence remains limited and inconsistent. The Aim: The primary objective was to evaluate the effects of permissive hypercapnia on inflammatory biomarkers in bronchoalveolar lavage fluid and serum during one-lung ventilation. The secondary objective was to assess the influence of different ventilation modes on these inflammatory responses. Methods: Forty patients undergoing elective lung surgery requiring OLV were prospectively enrolled and allocated to either a normocapnic (*n* = 20) or hypercapnic group (*n* = 20). BAL concentrations of TNF-α, IL-1β, IL-6, and IL-8 were measured before and after intervention. Serum IL-6, C-reactive protein (CRP), and leukocyte counts were also assessed. Outcome Measures: Primary outcome: Change in bronchoalveolar lavage (BAL) levels of preselected biomarkers for inflammation (TNF-α, IL-1β, IL-6, and IL-8) and in serum (IL-6, CRP, and WBC) between the normocapnic and hypercapnic groups during one-lung ventilation. Secondary outcome: Whether the inflammatory response differed according to the ventilation mode (pressure-controlled versus volume-controlled ventilation). Results: Baseline and post-intervention BAL concentrations of TNF-α, IL-1β, IL-6, and IL-8 were generally comparable between the hypercapnic and normocapnic groups. No significant differences were observed in post-intervention BAL concentrations of TNF-α, IL-1β, or IL-6 among the ventilated subgroups. However, post hoc analysis demonstrated significantly lower IL-8 concentrations in the hypercapnic-PC subgroup compared with the normocapnic-PC subgroup (*p* = 0.034). Within the normocapnic cohort, BAL IL-8 concentrations were significantly higher in the PC subgroup than in the VC subgroup (*p* = 0.012). In serum, postoperative IL-6 concentrations were significantly higher in the hypercapnic than in the normocapnic group (*p* = 0.003). Conclusions: Permissive hypercapnia was not associated with a reduction in BAL concentration of the pro-inflammatory cytokines TNF-α, IL-1β, IL-6, and IL-8. However, postoperative serum IL-6 concentrations were significantly higher in patients exposed to hypercapnia. As the duration of surgery, one-lung ventilation, and mechanical ventilation was longer in the hypercapnic group, the observed increase in serum IL-6 cannot be attributed solely to hypercapnia. Further large-scale randomized studies are required to clarify the immunological effects of permissive hypercapnia during thoracic surgery.

## 1. Introduction

Lung surgery represents a challenging field of thoracic surgery, encompassing a broad spectrum of procedures ranging from diagnostic interventions to complex resections, including lobectomy and pneumonectomy. Owing to the unique anatomical and physiological characteristics of the respiratory system, as well as the high prevalence of associated comorbidities, perioperative management of these patients requires a multidisciplinary approach [1].

One-lung ventilation (OLV) is an essential technique in thoracic anesthesia, providing optimal surgical exposure while simultaneously imposing substantial physiological stress on the patient. OLV is associated with ventilation-perfusion mismatch, impaired gas exchange, and an increased risk of hypoxemia. Furthermore, it contributes to the development of ventilator-induced lung injury (VILI), which may result in alveolar damage and activation of inflammatory pathways. These processes are accompanied by the release of pro-inflammatory cytokines, including IL-1β, IL-6, IL-8, and tumor necrosis factor-alpha (TNF-α), both locally within the lung parenchyma and systemically. Elevated inflammatory biomarker levels have been associated with an increased risk of postoperative complications and may reflect the severity of surgical and ventilatory stress. Excessive or prolonged inflammation may increase pulmonary vascular permeability, promote pulmonary edema, and contribute to lung injury. Prolonged OLV and suboptimal ventilation strategies may further exacerbate this inflammatory response, whereas lung-protective ventilation using low-tidal volumes and appropriate positive-end expiratory pressure (PEEP) has been shown to attenuate these effects [1].

Ventilator-induced lung injury (VILI) arises primarily from alveolar overdistension (volotrauma) and repetitive alveolar collapse and reopening (atelectrauma). These mechanisms disrupt the alveolo-capillary barrier, promote pulmonary edema, and stimulate the release of inflammatory mediators [2,3,4]. Consequently, lung-protective ventilation strategies have become an integral component of perioperative management in thoracic surgery. Such approaches typically involve the use of lower tidal volumes, limitation of airway pressures, and the application of appropriate levels of positive end-expiratory pressure (PEEP) to minimize mechanical stress and attenuate inflammatory responses [5].

However, lung protective ventilation often results in reduced minute ventilation and subsequent carbon dioxide retention, leading to hypercapnia [6]. Hypercapnia during general anesthesia may be classified as either permissive or therapeutic [7]. Permissive hypercapnia occurs as a consequence of reduced ventilator settings, whereas therapeutic hypercapnia involves the deliberate elevation of carbon dioxide levels through controlled ventilator strategies or exogenous carbon dioxide administration [8,9].

Interest has been directed toward the potential immunomodulatory effects of hypercapnia. Experimental studies suggest that elevated carbon dioxide levels may attenuate inflammatory response and reduce oxidative stress. However, these effects appear to be highly context-dependent, as prolonged hypercapnia may impair epithelial repair, alter host defense mechanisms, and delay recovery from lung injury [10,11,12,13,14]. Consequently, the role of hypercapnia during OLV remains controversial.

Since OLV may trigger both pulmonary and systemic inflammatory responses, further studies are needed to clarify the immunomodulatory effects of permissive hypercapnia in patients undergoing thoracic surgery.

### Study Objectives

Considering the evidence from the literature indicating that OLV may trigger inflammation and acute lung injury, strategies aimed at preventing or attenuating OLV-induced inflammation may be of critical importance for patients undergoing lung surgery. The protective strategy is lung-protective ventilation (i.e., low tidal volume and optimal PEEP), and permissive hypercapnia is the consequence of this strategy, with several previous studies demonstrating its efficacy and safety. Based on these findings, the primary objective was to evaluate the effects of permissive hypercapnia on inflammatory biomarkers in bronchoalveolar lavage fluid and serum during one-lung ventilation; the secondary objective was to assess whether different intraoperative ventilation modes (volume-controlled versus pressure-controlled ventilation) influence inflammatory biomarker responses in bronchoalveolar lavage fluid and serum during one-lung ventilation.

## 2. Materials and Methods

### 2.1. Study Design and Ethical Approval

This prospective clinical study was conducted between June 2025 and February 2026 at the University Clinical Center Niš. The study protocol was approved by the Ethics Committee of the University Clinical Center Niš (approval No 3856/8; date: 13 February 2025) and by the Ethics Committee of the Faculty of Medicine, University of Niš (approval No. 12-3102-1/2-2; date: 3 April 2025). Written informed consent was obtained from all participants before enrollment. The study was conducted in accordance with the principles of the Declaration of Helsinki.

### 2.2. Participants

A total of 40 adult patients scheduled for elective lung surgery requiring one-lung ventilation (OLV) were enrolled. Eligible participants were aged 18–85 years, classified as American Society of Anesthesiologists (ASA) Physical Status Classification I-III, scheduled for procedures expected to last at least 60 min, with anticipated OLV for the majority of the intraoperative period.

### 2.3. Exclusion Criteria

Patients were excluded if they met any of the following criteria: body mass index (BMI) > 35 kg/m^2^; impaired pulmonary function (vital capacity of forced expiratory volume in 1 s (FEV_1_) < 50% of predicted values); chronic infection; intracranial hypertension; prior intracranial hemorrhage; presence of active extrapulmonary malignancy; chronic pulmonary diseases (including chronic pneumonia, pneumoconiosis and silicosis); pulmonary hypertension; severe chronic obstructive pulmonary disease (COPD) with chronic hypercapnia; and patients undergoing bilobectomy or pneumonectomy.

### 2.4. Randomization and Study Groups

Randomization was performed using sequentially numbered, opaque, sealed envelopes. Patients were randomly allocated in a 1:1 ratio to the normocapnia (*n* = 20) or the hypercapnia group (*n* = 20).

The randomization sequence was generated by an independent statistician using variable block sizes of four and six and was concealed in sequentially numbered, opaque, sealed envelopes. Patient enrollment was performed by an anesthesiologist participating in the study, whereas the envelopes were opened immediately before induction of anesthesia by an investigator who was not involved in patient recruitment or perioperative management. Thus, allocation concealment was maintained until assignment.

The treating anesthesiologist was not blinded to the allocation.

### 2.5. Anesthetic Management

Following admission to the preoperative area, demographic and clinical data were recorded, including age, sex, height, body weight, ideal body weight (IBW), comorbidities, smoking status, and ASA physical status classification. An arterial catheter was inserted before induction of anesthesia to allow continuous invasive blood pressure monitoring and arterial blood gas analysis.

Patients were pre-oxygenated with 100% oxygen for 3–5 min before induction of anesthesia. General anesthesia was induced and maintained using target-controlled infusion (TCI) of propofol (Propofol-Lipuro^®^, B. Braun Melsungen AG, Melsungen, Germany) administered via an Alaris PK Syringe Pump (CreFusion, Basingstoke, UK) with a target effect-site concentration (Ceff) of 2.0–6.0 ng/mL (Schnider model) and remifentanil (Remifentanil Kabi^®^, Fresenius Kabi, Bad Homburg, Germany) with a target effect-site concentration (Ceff) of 2.0–8.0 ng/mL (Minto model). Neuromuscular blockade was achieved with rocuronium bromide (Esmeron^®^, Merck Sharp & Dohme B.V., Haarlem, The Netherlands) at a dose of 0.6 mg/kg. The depth of anesthesia was monitored using the Bispectral Index (BIS™ Monitoring System, Medtronic, Minneapolis, MN, USA) and wasmaintained between 40 and 60.

Following induction, all patients were intubated with a double-lumen endotracheal tube (Rüsch Bronchopart^®,^ Teleflex, Wayne, PA, USA). Correct placement of the tube was confirmed by fiberoptic bronchoscopy using a Broncho-Flex^®^ Single-Use Flexible Bronchoscope (Cook Medical, Bloomington, IN, USA) immediately after intubation and reassessed following lateral positioning.

### 2.6. Ventilation Protocol

There were two ventilation protocols: volume-controlled (VCV) mode and pressure-controlled (PCV) mode both performed using a Dräger Perseus A500 anesthesia workstation (Drägerwerk AG & Co. KGaA, Lübeck, Germany). The choice of modes was made according to a predefined institutional ventilation protocol and was not part of the randomized process.

During two-lung ventilation, patients in the VC group received a tidal volume of 7 mL/kg IBW. During one-lung ventilation OLV, tidal volume was reduced to 5 mL/kg IBW. For patients ventilated using the PC mode, inspiratory pressure was adjusted to achieve equivalent tidal volumes. PEEP was initially set at 5 cmH_2_O and remained unchanged unless hypoxemia occurred. The fraction of inspired oxygen (FiO_2_) was initially set at 1.0 and reduced to 0.5 after initiation of OLV. The intended strategy was to minimize FiO_2_ at 0.5; however, FiO_2_ was increased when significant hypoxemia persisted for several minutes. Prior to and following the initiation of OLV, an alveolar recruitment maneuver was applied (manual inflations for 10–40 s to achieve a peak airway pressure of 30–40 cmH_2_O).

In cases of severe hypoxemia (<88%), the first intervention was an increase in the inspired oxygen fraction (FiO_2_). If adequate oxygenation was not achieved, further ventilatory adjustments were implemented, including increases in PEEP and inspiratory time, as well as application of the recruitment maneuver according to the clinical situation and ventilatory parameters.

Respiratory rate was adjusted to maintain the predefined PaCO_2_ targets:

- Normocapnic group: 35–45 mmHg;
- Hypercapnic group: 55–75 mmHg.

An inspiratory-to-expiratory (I:E) ratio ranging from 1:2 to 1:1 was maintained throughout the procedure. Throughout the surgical procedure, patients were monitored using a 5-lead electrocardiogram (ECG), invasive blood pressure measurement, heart rate, and depth of anesthesia using the Bispectral Index (BIS).

Ventilatory parameters, including tidal volume (TV), fraction of inspired oxygen (FiO_2_), respiratory rate (RR), SpO_2_, expired carbon dioxide (EtCO_2_), PEEP, peak (Ppeak), inspiratory pressure above PEEP (Pinsp), plateau airway pressure (Pplat), driving pressure (ΔP)—calculated as the difference between Pplat and PEEP), and the dynamic compliance, together with arterial blood gas parameters, were assessed at predefined time points.

In the VC subgroup, Ppeak and Pplat were recorded. In the PC subgroup, the set inspiratory pressure above PEEP (Pinsp) was recorded, as it represented the target inspiratory airway pressure. In PC mode, Pinsp was used as a surrogate for inspiratory airway pressure under controlled mechanical ventilation.

The predefined time points were: prior to anesthesia induction (T_bas_), after induction, after intubation and initiation of two-lung ventilation (T_1_), after positioning in the lateral decubitus position (T_2_), 15 min from the onset of OLV (T_3_), 1 h after the onset of OLV (T_4_), and after conversion back to two-lung ventilation in the supine position (T_5_).

### 2.7. Bronchoalveolar Lavage Fluid and Biomarker Analysis

Prior to initiation of one-lung ventilation and following the return to two-lung ventilation and lung re-expansion, bronchoalveolar lavage (BAL) fluid was collected from two bronchopulmonary segments (the same segments before and after intervention) of both the collapsed and ventilated lungs. Samples from both lungs were pooled and analyzed together. BAL was performed using a fiberoptic bronchoscope and 60 mL of sterile saline, with gentle aspiration under negative pressure of –50 cmH_2_O. The recovered lavage fluid was collected, and 10–15 mL of BAL fluid was obtained for laboratory analysis. Due to variability in recovery volume, BAL samples were analyzed without normalization to the initial instilled lavage volume. BAL fluid was centrifuged at 1500 rpm for 15 min at room temperature. The supernatant was frozen and stored at –80 °C, and interleukin levels were determined by enzyme-linked immunosorbent assay (ELISA) with commercially available kits (FineTest, Wuhan, China China) according to the manufacturer’s instructions. All samples were analyzed in the same assay run. The detection limits of the kits were as follows: TNF –α 3.8 pg/mL, IL-1β 1.56 pg/mL, IL-6 2.5 pg/mL, and IL-8 7.8 pg/mL.

Peripheral venous blood samples (10 mL) were obtained at the beginning and end of surgery for the measurement of serum leukocyte count (WBC), C-reactive protein (CRP) and IL-6 levels. At the end of the procedure, all patients were extubated in the operating room and transferred either to the intensive care unit (ICU) or to the surgical ward based on the clinician’s judgment.

Perioperative blood samples and BAL fluid samples were analyzed at the Central Laboratory of the University Clinical Center Niš and the Laboratory for Functional Genomics and Proteomics, Scientific Research Center for Biomedicine, Faculty of Medicine, University of Niš.

The duration of the surgery and anesthesia, duration of OLV, intraoperative fluid administration, urine output, and intraoperative blood loss were recorded. Potential adverse effects associated with hypercapnia were monitored as immediate postoperative complications, and included postoperative visual disturbances, the occurrence of tachycardia and arrhythmias, postoperative emotional dysfunction, agitation, disorientation, panic symptoms, and convulsive seizures.

No protocol deviations were recorded during the study period.

### 2.8. Outcomes

The primary outcome of the study was to evaluate the effect of permissive hypercapnia on the inflammatory response during one-lung ventilation by comparing a prespecified panel of inflammatory biomarkers between the normocapnic and hypercapnic groups. The primary outcome measures included BAL concentrations of TNF-α, IL-1β, IL-6, and IL-8, together with serum IL-6, CRP, and WBC, measured at predefined sampling time points.

The secondary outcome was to evaluate whether the inflammatory response differed according to the ventilation mode (pressure-controlled vs. volume-controlled ventilation), and these subgroup analyses should be interpreted as exploratory.

### 2.9. Study Power and Sample Size

An online sample size calculator was used to estimate the representative sample size https://statulator.com/SampleSize/ss2PM.html. accessed on 20 November 2024 The initial assumptions included a statistical power of 80% and a type I error rate of 0.05 for two-tailed hypothesis testing, which yielded an estimated sample size of 34 patients. To compensate for the potential attrition during the study, the sample size was increased by 15%, resulting in a total planned enrollment of 40 patients.

Statistical analysis was performed using IBM SPSS Statistics software (version 26.0, IBM Corp., Armonk, NY, USA). Continuous variables were tested for normality using the Shapiro–Wilk test and visual inspection of histograms and Q–Q plots. All variables were presented as mean ± standard deviation (SD). Categorical variables were summarized as absolute numbers and percentages.

Analysis was performed using RM-ANOVA with two time points (baseline and post-intervention) as a within-subject factor and group (normocapnia vs. hypercapnia) as a between-subject factor. A group × time interaction was used as the primary test of treatment effect on change. F-test values, *p*-values and effect size (partial η^2^) are shown. Mauchly’s test was used to assess sphericity, and when the assumption of sphericity was violated, Greenhouse–Geisser correction was applied.

Secondary and exploratory analyzes were performed using the Mann–Whitney test, Kruskal–Wallis test for comparison of continuous variables and the chi-square test for categorical variables. These results are interpreted as exploratory only.

To further assess independent predictors of postoperative inflammatory response, exploratory univariate and multivariate linear regression analyses were performed with postoperative serum IL-6 levels as the dependent variable. Variables of clinical relevance were entered into separate regression models. Regression results were expressed as standardized coefficients (β), unstandardized coefficients (B), 95% confidence intervals (95% CI), and *p*-values. All statistical tests were two-tailed, and *p*-values <0.05 were considered statistically significant. The Holm–Bonferroni procedure was used to control multiple comparisons.

The study was conducted and reported in accordance with the CONSORT guidelines. Trial registration was not required by the local Ethics Committee.

## 3. Results

**Figure 1:**
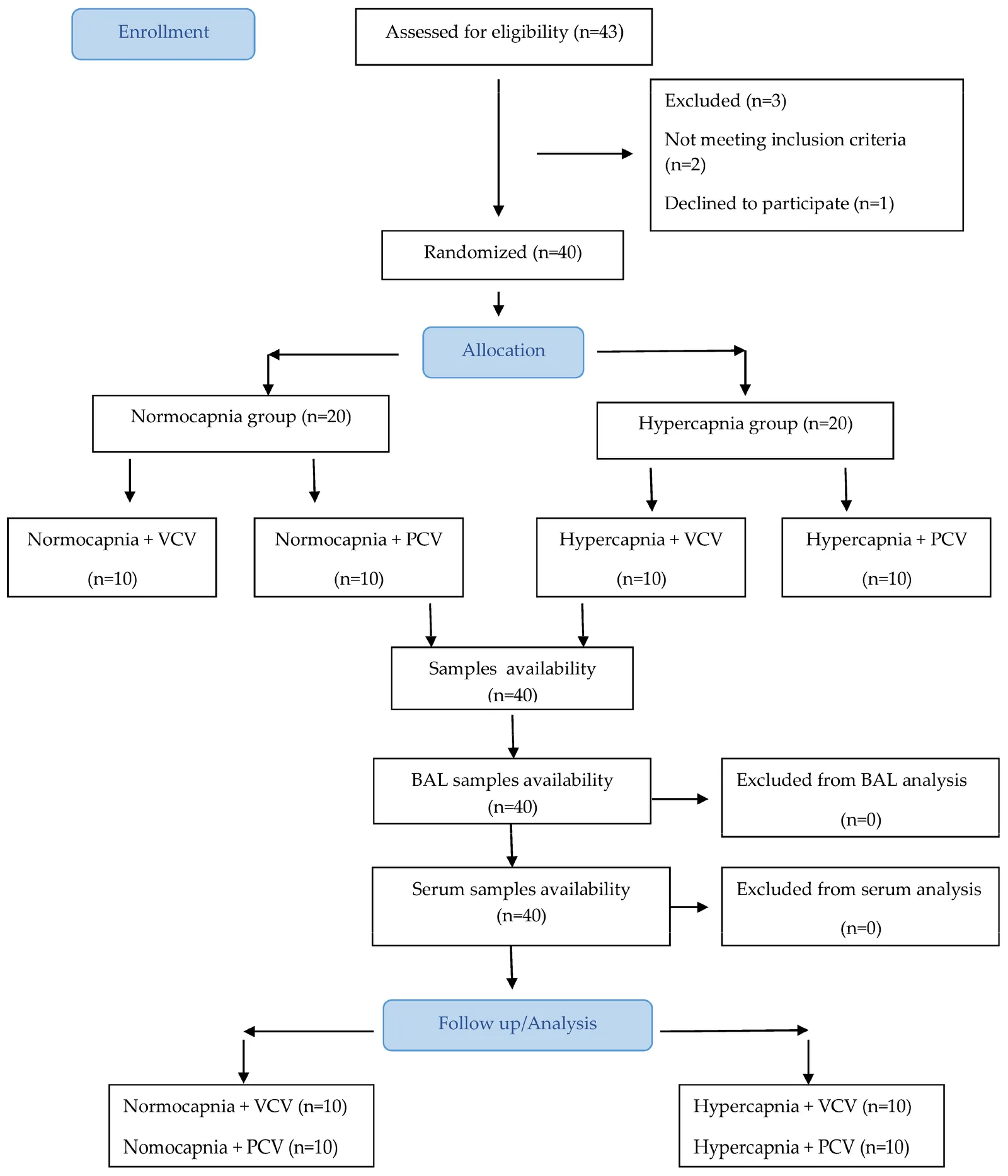
CONSORT flowchart.

A total of 40 patients were enrolled and completed the study (Figure 1). No participants were excluded after randomization, and no protocol deviations were recorded.

The hypercapnic and normocapnic groups were generally comparable with respect to demographic and baseline characteristics. No statistically significant differences were observed in age, sex distribution, anthropometric measurements, ASA physical status, or the prevalence of major comorbidities including hypertension, chronic obstructive pulmonary disease, and diabetes mellitus. Mean age was the same in both groups (65 years). The majority of patients in both groups were classified as ASA III (in the hypercapnic group n = 13, in the normocapnic group n = 11). The most common comorbidities were hypertension (HTA), diabetes mellitus (DM) and chronic obstructive pulmonary disease (COPD). The hypercapnic group included more smokers (n = 15 vs. n = 8) and a higher proportion of thoracotomies (n = 11 vs. n = 5). The duration of surgery was shorter in the normocapnic group (103.35 ± 68.31 min), while in the hypercapnic group was 132.90 ± 66.63 min. Similarly, the duration of OLV was longer in the hypercapnic group (112.70 ± 67.18 min vs. 88.10 ± 54.50 min), as well as the duration of mechanical ventilation (182.00 ± 75.88 min vs. 148.40 ± 68.43 min) (Table 1).

The two groups were well balanced with respect to demographic and baseline clinical characteristics, with no statistically significant differences in age (*p* = 0.997), sex distribution (*p* = 0.519), anthropometric measures, ASA physical status (*p* = 0.413), or comorbidities, including hypertension (*p* = 0.490), chronic obstructive pulmonary disease (*p* = 0.723), and diabetes mellitus (*p*= 0.429) (Table 1).

The proportion of smokers was significantly higher in the hypercapnic group than in the normocapnic group (75.0% vs. 40.0%, *p* = 0.028) (Table 1).

Mean perioperative PaCO_2_ values were significantly higher in the hypercapnic group (59.11 ± 6.50 mmHg vs. 49.53 ± 0.505 mmHg, *p* < 0.001), confirming achievement of the predefined PaCO_2_ targets. No significant differences were observed in PaO_2_ values (*p* = 0.420) (Table 1).

Surgical characteristics, including operative approach (*p* = 0.117), side of surgery (*p* = 0.774), duration of surgery (*p* = 0.174), duration of one-lung ventilation (*p* = 0.965), intraoperative fluid administration (*p* = 0.704), blood loss (*p* = 0.055), duration of mechanical ventilation (*p* = 0.091), and urine output (*p* = 0.964), did not differ significantly between the groups (Table 1). However, longer operative duration and greater intraoperative blood loss were observed in the hypercapnic group, although these differences did not reach statistical significance.

Mean airway pressures and respiratory mechanics remained comparable between groups throughout the perioperative period (Table 2).

No statistically significant differences were observed in peak inspiratory pressure, plateau pressure, driving pressure, or dynamic compliance (all *p* > 0.05), indicating that respiratory mechanics were comparable between the hypercapnic and normocapnic groups throughout the study period (Table 2).

There was a significant interaction between time x group for BAL concentration of IL-6 (*p* = 0.029) (Table 3). Post hoc analysis revealed significant differences in IL-6 concentration between the two measurements in the normocapnic group (*p* = 0.010, effect size = 0.790). All other inflammatory markers did not significantly change, as reported by rmANOVA. However, after applying Holm–Bonferroni correction, the significant interaction no longer remained (*p* = 0.162).

In exploratory subgroup analyses, when patients were stratified according to ventilation mode, no significant differences were observed among the four subgroups in post-intervention BAL concentrations of IL-6, TNF -α, or IL-1β (Table 4). In contrast, a significant difference was observed in post-intervention BAL IL-8 concentrations (χ^2^ = 8788; *p* = 0.032). Post hoc analysis demonstrated significantly lower IL-8 levels in the hypercapnic–PCV subgroup than in the normocapnic–PCV subgroup (15.59 ± 5.46 vs. 59.50 ± 43.97; Z = 2.121; *p* = 0.032) (Supplementary Table S1).

For WBC, mixed-design ANOVA showed a statistically significant effect of time (*p* < 0.001) and Group × Timeinteraction (*p* = 0.007); both effects remained statistically significant after Holm–Bonferroni correction (p_holm = 0.007 for both), while the group effect was not significant (*p* = 0.469; p_holm = 1.000) (Table 4). For IL-6, a statistically significant effect of group (*p* = 0.002) and time (*p* < 0.001) was observed, which remained significant even after correction (p_holm = 0.014 and p_holm = 0.007, respectively); however, the Group × Time interaction, significant in the uncorrected analysis (*p* = 0.027), did not retain significance after Holm–Bonferroni correction (p_holm = 0.162), indicating that the change in this marker over time was not statistically different between groups.

Subgroup analysis revealed a significant difference in postoperative IL-6 concentrations (*p* = 0.006). Patients receiving pressure-controlled ventilation within the hypercapnic group exhibited significantly higher postoperative IL-6 levels than in patients receiving pressure-controlled ventilation within the normocapnic group (65.40 ± 48.24 vs. 22.11 ± 20.79, *p* = 0.019), and the VC normocapnic group (*p* =0.001), as confirmed by the Mann–Whitney test (Supplementary Table S2).

In univariable linear regression analyses, assignment to the hypercapnic group was significantly associated with postoperative serum IL-6 concentrations (β = −0.456, *p* = 0.003). In addition, longer durations of surgery (β = 0.559, *p* < 0.001), one-lung ventilation (β = 0.662, *p* < 0.001), and mechanical ventilation (β = 0.590, *p* < 0.001) were all significantly associated with increased postoperative IL-6 levels.

Smoking status, intraoperative blood loss, and surgical approach were not significantly associated with postoperative IL-6 concentrations, although a trend toward higher IL-6 values wasobserved among smokers and patients undergoing thoracotomy (*p* = 0.068).

These findings suggest that both hypercapnia and prolonged perioperative exposure, particularly longer surgical and ventilation durations, may contribute to postoperative systemic inflammatory response (Table 5 and Table 6).

Exploratory multivariable analyses demonstrated that the association between group allocation and postoperative IL-6 remained significant after adjustment for the duration of one-lung ventilation and mechanical ventilation. Each of these perioperative variables also remained independently associated with postoperative serum IL-6 concentrations.

The PaO_2_/FiO_2_ ratio decreased in both groups following initiation of OLV and subsequently improved toward the end of the procedure.

As expected, PaCO_2_ values remained consistently higher in the hypercapnic group throughout the study period. A gradual increase was observed after initiation of OLV, with peak values occurring approximately one hour after OLV commencement.

**Figure 2:**
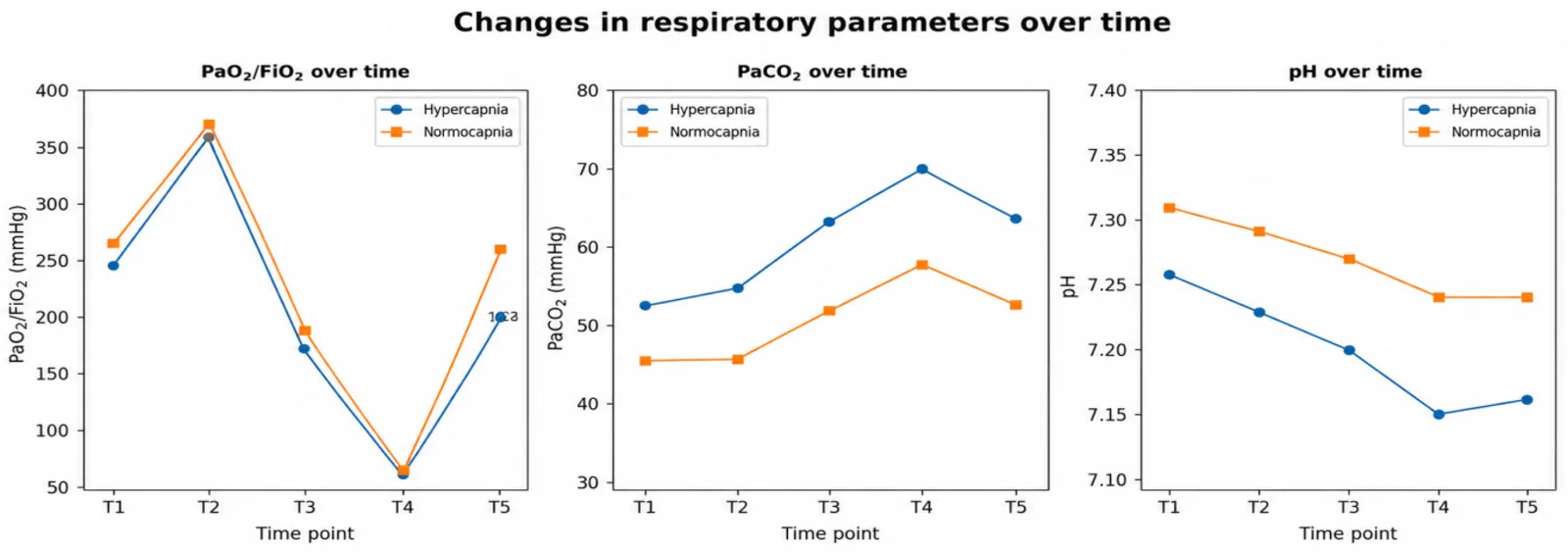
Changes in PaO/FiO_2_ ratio, PaCO_2_, and pH over time in the Hypercapnic and Normocapnic groups.

Arterial pH values decreased over time in both groups and reached lower values in the hypercapnic group, reflecting the physiological consequence of permissive hypercapnia (Figure 2. Changes in PaO/FiO_2_ ratio, PaCO_2_, and pH over time in the Hypercapnic and Normocapnic groups). No buffering agents were administered during the study.

No statistically significant differences were observed between the hypercapnic and normocapnic groups regarding intraoperative and immediate postoperative complications (*p* = 0.121; Table 7).

Decreased oxygen saturation was the most common intraoperative complication. Although its incidence differed numerically between the study groups, the difference was not statistically significant (Table 7).

Among immediate postoperative complications, agitation occurred at similar frequencies across all groups (*p* = 0.782) (Table 7).

## 4. Discussion

The present study evaluated the effects of permissive hypercapnia on local and systemic inflammatory responses in patients undergoing thoracic surgery with one-lung ventilation (OLV). The principal findings were that permissive hypercapnia did not significantly affect BAL fluid concentrations of TNF-α, IL-1β, IL-6, or IL-8 compared with normocapnia.

Although BAL IL-6 concentrations appeared to demonstrate a different temporal pattern between the study groups in the initial repeated-measures analysis, this finding lost statistical significance after correction for multiple comparisons. These results suggest that permissive hypercapnia did not exert a consistent effect on local pulmonary IL-6 levels during OLV. Interestingly, a significant within-group increase in BAL IL-6 was observed only in the normocapnic group. However, because the interaction did not remain significant after Holm–Bonferroni correction, this observation should be interpreted cautiously and cannot be considered evidence of a true between-group difference (Table 3). In addition, lower BAL IL-8 concentrations were observed in patients receiving pressure-controlled ventilation combined with permissive hypercapnia, suggesting a potential interaction between ventilation mode and the pulmonary inflammatory response (Supplementary Table S1). These findings should be interpreted in the context of the complex pathophysiological mechanisms associated with one-lung ventilation, which may influence both local and systemic inflammatory responses.

One-lung ventilation remains an essential component of modern thoracic anesthesia but is associated with substantial physiological and inflammatory challenges. Ventilation–perfusion mismatch, cyclic alveolar collapse and reopening, mechanical stress, and oxidative injury contribute to pulmonary inflammation and may increase the risk of postoperative pulmonary complications (PPCs). Consequently, considerable efforts have been directed toward identifying ventilation strategies that attenuate these detrimental effects while preserving adequate gas exchange.

Earlier mechanical ventilation strategies relied on high tidal volumes (up to 15 mL/kg) to prevent atelectasis; however, particularly during OLV, such approaches were later recognized as major contributors to ventilator-induced lung injury (VILI). Therefore, lung-protective ventilation has become the standard of care during thoracic surgery. This strategy incorporates lower tidal volumes (4–6 mL/kg predicted body weight), moderate inspired oxygen fractions (FiO_2_ 0.5–0.6), the application of PEEP (5–10 cmH_2_O or individualized) and limitation of plateau pressure (<30 cmH_2_O) [6,15]. Respiratory rate is adjusted to maintain normocapnia (PaCO_2_ = 35–45 mmHg) [16] while the target oxygen saturation should be ≥88% [17].

These strategies reduce cyclic alveolar collapse and reopening, mitigating atelectrauma and development of VILI [11,18,19]. More recently, the iOLA trial further strengthened the evidence supporting individualized lung-protective ventilation. In this large multi centre randomized controlled trial, the intraoperative open-lung approach (iOLA), incorporating recruitment maneuvers, individualized PEEP, and tailored postoperative respiratory support, significantly reduced the incidence of severe postoperative pulmonary complications compared with conventional lung-protective ventilation [20]. Further evidence regarding the role of lung-protective ventilation comes from the PROTHOR trial [21]. In contrast to the iOLA study, the PROTHOR trial did not demonstrate a significant reduction in PPCs when comparing a lung-protective ventilation strategy with conventional management. However, both studies confirmed the feasibility and safety of protective ventilation approaches, including the use of lower tidal volumes and individualized PEEP strategies without increased risk of extrapulmonary complications.

Hypercapnia, which may occur during lung-protective ventilation, may be categorized as permissive or therapeutic. Permissive hypercapnia represents the most common clinical scenario; however, its physiological effects remain incompletely understood. Accumulating evidence suggests that elevated CO_2_ levels may exert potential protective effects through modulation of the oxygen delivery-consumption relationship, attenuation of oxidative stress, and reduction in the release of pro-inflammatory mediators [22,23,24]. These mechanisms may contribute to organ protection in conditions associated with ischemia and oxidative injury.

In contrast, therapeutic hypercapnia refers to the deliberate induction of elevated CO_2_ concentrations with the aim of achieving potential protective or immunomodulatory effects. Notably, both permissive and therapeutic hypercapnia have been investigated in conditions such as ARDS, neonatal respiratory distress, and acute severe asthma, with experimental and clinical data suggesting potential reduction in pulmonary inflammation and oxidative stress [25]. Experimental studies suggest that hypercapnic acidosis may attenuate lung injury by reducing pulmonary edema, alveolar-capillary permeability, and pro-inflammatory mediator production, while improving respiratory mechanics. However, these beneficial effects have not been consistently confirmed in clinical studies, highlighting the need for further investigation [26].

Lung injury during OLV is mediated by multiple mechanisms including oxidative stress, alveolar overdistension, repetitive alveolar collapse and reopening, vascular compression, and increased pulmonary vascular resistance. These processes are accompanied by alterations in cytokine expression patterns, particularly involving TNF-α, IL-1β, IL-6, IL-8, and IL-10 [9].

Interleukins serve as early markers of inflammatory activation. IL-6 plays a central role in the acute-phase response and CRP synthesis [27,28], and elevated perioperative IL-6 levels have been associated with postoperative complications, including pulmonary complications (PPCs) following thoracic surgery. IL-8 promotes neutrophil recruitment and pulmonary inflammation [29], whereas IL-10 reflects anti-inflammatory activity and immune regulation [30]. TNF-α contributes to endothelial dysfunction and increased capillary permeability, and together with IL-6 and IL-8, has been associated with the development of PPC [31,32].

The earliest evidence supporting the potential protective role of hypercapnic acidosis in VILI originated from experimental studies. Broccard et al. [33] demonstrated, in an isolated rabbit lung model, that hypercapnia during injurious ventilation attenuated pulmonary edema, microvascular permeability, and protein leakage into BAL fluid. Subsequent experimental studies further suggested immunomodulatory effects of therapeutic hypercapnia, including reduced neutrophil recruitment, decreased synthesis of pro-inflammatory mediators (TNF-α, IL-6, IL-8), lower systemic inflammatory marker levels and increased anti-inflammatory IL-10 concentrations [34].

Additional experimental studies have demonstrated potential beneficial effects of hypercapnia on respiratory mechanics, including reduced airway pressures and improved lung compliance [14,35]. During OLV, hypercapnia may mitigate reductions in compliance and increases in airway resistance through bronchodilatatory and anti-inflammatory mechanisms [14,25]. Additionally, hypercapnia may improve oxygenation by increasing the PaO_2_/FiO_2_ ratio, potentially mediated by the Bohr effect and rightward shift in the oxyhemoglobin dissociation curve.

However, not all experimental findings are favorable. Several animal studies have suggested that hypercapnia may impair epithelial repair and alveolar recovery following VILI, primarily through inhibition of NF-κB-dependent cellular migration and reduced alveolar edema clearance mediated by Na^+^/K^+^-ATPase dysfunction [14,36,37]. Notably, these effects appear to be independent of pH and may be mediated by signaling pathways involving cytoskeletal proteins that act as CO_2_ sensors [38,39,40,41,42].

Furthermore, some experimental studies have reported potential adverse effects of hypercapnia, including increased oxidative stress and markers of lung injury [43,44]. Lang et al. [43] demonstrated that hypercapnic ventilation was associated with higher BAL protein concentrations, an increased wet-to-dry lung weight ratio, and greater inflammatory cell infiltration. These findings suggest that the effects of hypercapnia may depend on the magnitude of CO_2_ elevation, the duration of exposure, and the underlying ventilatory conditions [42].

Hypercapnia has also been associated with impaired host defense mechanisms in experimental infection models. In murine models of *Pseudomonas aeruginosa* pneumonia, exposure to elevated CO2 concentrations resulted in higher mortality, increased bacterial colony counts, and suppression of pulmonary IL-6 and TNF-α production, suggesting potentially significant immunosuppressive effects [44].

Clinical studies investigating permissive hypercapnia during OLV have yielded inconsistent results. Although some studies have reported attenuation of inflammatory responses, others have failed to demonstrate clinically relevant anti-inflammatory effects. In the present study, permissive hypercapnia was not associated with significant changes in BAL concentrations of TNF-α, IL-1β, IL-6, or IL-8 compared with normocapnia (Table 3), suggesting no substantial reduction in local pulmonary inflammation. These findings support the concept that anti-inflammatory effects observed in experimental models may not be fully reproduced in the complex perioperative setting of thoracic surgery.

Interestingly, subgroup analysis revealed significantly lower BAL IL-8 concentrations in patients receiving pressure-controlled ventilation combined with permissive hypercapnia, consistent with previously reported findings [9,20,21,22,31,32] (Supplementary Table S1). IL-8 plays a central role in neutrophil recruitment and activation and is considered a sensitive marker of pulmonary inflammatory activity. The reduction observed in this subgroup may indicate a localized anti-inflammatory effect of hypercapnia that was not detectable through analysis of other cytokines. However, given the relatively small sample size and exploratory nature of the subgroup analysis, this finding should be interpreted with caution and requires confirmation in larger studies.

The immunomodulatory effects of hypercapnia have been extensively investigated. Experimental and clinical evidence suggests that hypercapnia can modulate both innate and adaptive immune responses by reducing the release of pro-inflammatory cytokines, including IL-6, TNF-α, and IL-1β [14,45]. Additionally, hypercapnia has been shown to impair the phagocytic capacity of alveolar macrophages in both experimental and clinical settings [14,46,47]. These effects may be mediated through inhibition of the canonical NF-κB pathway, a key regulator of inflammatory gene expression, potentially promoting anti-inflammatory signaling [14,48,49].

However, it remains unclear whether these effects are mediated by hypercapnia, acidosis, or their interaction. While extracellular acidosis may enhance inflammation through neutrophil activation and delayed apoptosis, hypercapnic acidosis has also been shown to inhibit xanthine oxidase activity, potentially reducing ischemia–reperfusion injury [34,50]. Laffey et al. [51] suggested that acidosis itself may contribute substantially to the protective effects observed in experimental studies.

Although baseline and overall WBC values in serum did not differ between groups, leukocyte counts exhibited significantly different temporal changes, suggesting that permissive hypercapnia influenced the postoperative systemic leukocyte response (Table 4).

In contrast to the BAL findings, postoperative serum IL-6 concentrations were significantly higher in the hypercapnic group (Table 4). IL-6 is a key mediator of the systemic inflammatory response and has been associated with surgical trauma, tissue injury, and postoperative complications. At first glance, this observation appears inconsistent with the proposed anti-inflammatory properties of hypercapnia. However, several important factors should be considered when interpreting this result.

The surgical approach and the magnitude of operative stress may have contributed to the observed differences. Yim et al. [52] reported lower postoperative IL-6 and IL-8 levels in patients undergoing VATS compared with those undergoing an open thoracic approach. In our study, patients in the hypercapnic group had longer surgical duration, prolonged one-lung ventilation, and longer mechanical ventilation duration (Table 5 and Table 6). Each of these factors has been associated with increased inflammatory activation and may have contributed to the observed higher postoperative serum IL-6 concentrations observed in this group. These findings suggest that mechanical stress, even in the absence of severe structural lung injury, may initiate molecular pathways of inflammation through mechantransduction mechanisms [53]. Furthermore, these subgroup comparisons should be interpreted cautiously, as the study was not powered to detect differences between subgroups.

Regression analyses further supported this interpretation. In addition to group allocation, duration of surgery, duration of one-lung ventilation, and duration of mechanical ventilation were independently associated with postoperative IL-6 concentrations. These results suggest that the systemic inflammatory response observed in the hypercapnic group may reflect the cumulative impact of prolonged perioperative exposure rather than a direct detrimental effect of hypercapnia itself.

Smoking status and intraoperative blood loss were not significantly associated with postoperative IL-6 levels, although both demonstrated a trend toward higher IL-6 concentrations. Similarly, patients undergoing thoracotomy tended to have higher postoperative IL-6 levels compared with those undergoing VATS, but the association did not reach statistical significance (Table 5).

The immunological effects of hypercapnia remain incompletely understood. Although elevated CO_2_ levels may modulate inflammatory responses through NF-kB signaling and cytokine regulation,, prolonged hypercapnia may also impair epithelial repair and host defense mechanisms. These findings suggest that the effects of hypercapnia are highly dependent on its magnitude, duration, and clinical context [54]. In our study, permissive hypercapnia was not associated with an increased incidence of postoperative complications or serious adverse events, supporting its feasibility and safety under controlled perioperative conditions. Blood biomarkers may provide additional insight into inflammatory activity and prediction of postoperative complications; however, their clinical relevance requires further investigation beyond the scope of this study [55].

The immediate postoperative complications evaluated in this study were selected based on their reported association with intraoperative permissive hypercapnia. Although agitation was most frequently observed, its incidence did not differ significantly between the study groups (Table 7).

Overall, our findings suggest that permissive hypercapnia during thoracic surgery with OLV does not exert a consistent anti-inflammatory effect at the pulmonary level, although its interaction with ventilation strategy may influence specific inflammatory pathways. He observed that an increase in systemic IL-6 appears to be multifactorial and associated with the cumulative perioperative inflammatory burden rather than hypercapnia alone.

It should be emphasized that most experimental studies demonstrating anti-inflammatory effects of hypercapnia have been conducted in models of pre-existing lung injury. In contrast, evidence regarding the effects of hypercapnia in relatively healthy lungs remained limited, and further studies are required to clarify whether the observed immunomodulatory effects are dependent on the underlying pulmonary condition.

## 5. Conclusions

Permissive hypercapnia during OLV was not associated with a significant reduction in BAL fluid concentrations of the pro-inflammatory cytokines TNF-α, IL-1β, IL-6, and IL-8. However, lower BAL IL-8 concentrations were observed in patients receiving pressure-controlled ventilation combined with permissive hypercapnia, suggesting a potential interaction between ventilation mode and the local inflammatory response.

Postoperative serum IL-6 concentrations were significantly higher in the hypercapnic group. This finding should be interpreted with caution, as longer durations of surgery, one-lung ventilation, and mechanical ventilation may have contributed substantially to the observed systemic inflammatory response. Therefore, no definitive local anti-inflammatory effect was demonstrated; the systemic IL-6 findings require further confirmation, and subgroup analyses should be considered exploratory and hypothesis-generating.

Overall, permissive hypercapnia appears to be a feasible and clinically safe ventilation strategy during thoracic surgery. Further adequately powered multicenter randomized controlled trials are required to clarify its immunomodulatory effects and potential clinical benefits during one-lung ventilation.

### 5.1. Strengths and Limitations

This study has several strengths. It was conducted prospectively, incorporated both local and systemic inflammatory biomarkers, and evaluated the interaction between permissive hypercapnia and different ventilation modes during OLV.

Several limitations should be acknowledged. First, the relatively small sample size may have limited the ability to detect modest differences in inflammatory biomarkers between groups. Second, the single-center design may restrict the generalizability of the findings to other patient populations and clinical settings. Third, although randomization was performed, differences in operative duration, duration of one-lung ventilation, and duration of mechanical ventilation may have influenced postoperative inflammatory responses and represented potential confounding factors. Additionally, subgroup analyses according to ventilation mode were exploratory and limited by the small number of patients in each subgroup; therefore, these findings should be considered hypothesis-generating. Fourth, inflammatory mediators were assessed at predefined perioperative time points. Additional postoperative measurements could have provided a more comprehensive understanding of cytokine kinetics and inflammatory dynamics. Furthermore, only selected inflammatory biomarkers were analyzed; inclusion of additional markers related to immune activation, oxidative stress, and endothelial injury could have provided a broader characterization of the biological effects of permissive hypercapnia.

Finally, the absence of prospective clinical trial registration represents a limitation of the present study. Although the study was conducted prospectively according to a predefined protocol, approved by the institutional Ethics Committee, and performed in accordance with ethical standards, prospective registration of randomized controlled trials is increasingly recommended to enhance transparency and reduce the risk of selective reporting. Future investigator-initiated studies should be prospectively registered in internationally recognized trial registries whenever feasible.

### 5.2. Practical Implications

The findings of the present study suggest that permissive hypercapnia should not be expected to produce a substantial reduction in pulmonary inflammatory cytokine concentrations during one-lung ventilation. However, the absence of major adverse effects and the potential reduction in IL-8 concentrations observed in selected subgroups indicate that permissive hypercapnia may remain a reasonable component of lung-protective ventilation strategies in appropriately selected patients.

## Figures and Tables

**Table 1:** Demographic and surgical characteristics of patients in the normocapnic and hypercapnic group.

Characteristics	Hypercapnic Group n = 20	Normocapnic Group n = 20	*p*-Value
Sex male	13 (65.0%)	11 (55.0%)	0.519
Age (years)	64.90 ± 9.27	64.80 ± 12.30	0.997
Height (cm)	170.55 ± 11.05	170.50 ± 9.15	0.988
Weight (kg)	73.95 ± 13.29	71.35 ± 10.24	0.493
Ideal weight (kg)	64.85 ± 11.56	64.45 ± 9.95	0.907
ASA classification			
1	1 (5.0%)	0 (0.0%)	
2	6 (30.0%)	9 (45.0%)	
3	13 (65.0%)	11 (55.0%)	0.413
HTA	15 (75.0%)	13 (65.0%)	0.490
COPD	6 (30.0%)	5 (25.0%)	0.723
DM	5 (25.0%)	3 (15.0%)	0.429
Smoking status	15 (75.0%)	8 (40.0%)	0.028
Thoracotomy/VATS	11 (55.0%)/9 (45.0%)	5 (25%)/15 (75%)	0.053
Right side of the operation: Collapsed lung	12 (60.0%)	13 (65.0%)	0.744
PaO_2_	240.85 ± 93.01	264.45 ± 90.03	0.420
PaCO_2_	59.11 ± 6.50	49.53 ± 5.05	<0.001
Duration of surgery (min)	166.27 ± 48.94	184.01 ± 44.25	0.174
Duration of one lung ventilation (min)	112.70 ± 67.18	88.10 ± 54.50	0.965
Total volume of fluids infused (ml)	1600.0 ± 347.92	1550.00 ± 394.03	0.704
Blood loss (ml)	160.00 ± 138.22	92.50 ± 100.36	0.055
Duration of mechanical ventilation (min)	182.00 ± 75.88	148.40 ± 68.43	0.091
Diuresis (ml)	418.42 ± 242.22	457.89 ± 348.91	0.964

**Table 2:** Average pressure values and compliance during the operative period (T_1_–T_5_).

	Hypercapnic Group	Normocapnic Group	*p*-Value
* Peak Inspiratory Pressure (Ppeak) (cmH_2_O)	15.03 ± 2.79	15.99 ± 2.60	0.433
Plateau pressure(Pplat) (cmH_2_O)	14.99 ± 2.72	15.58 ± 2.59	0.486
* Inspiratory Pressure(Pinsp)	14.66 ± 3.01	15.96 ± 2.39	0.299
Dynamic compliance (mL/cmH_2_O)	50.59 ± 21.19	50.75 ± 14.70	0.665

* Peak Inspiratory pressure—(Ppeak)-average value for both subgroups (VC and PC mode); * Inspiratory pressure (Pinsp)-average value for PC group.

**Table 3:** Inflammatory markers in BAL.

Marker	Group	Before		After		rmANOVA Analysis			
		N	Mean ± SD	N	Mean ± SD		Group	Time	Group: Time
IL6	Hypercapnic	13	2.09 ± 2.20	17	1.97 ± 1.88	*p*-value	0.142	0.030	0.029
	Normocapnic	11	0.86 ± 1.21	15	6.62 ± 9.88	effect size	0.057	0.128	0.130
TNF	Hypercapnic	13	16.75 ± 29.76	17	14.52 ± 13.01	*p*-value	0.121	0.190	0.149
	Normocapnic	14	18.20 ± 16.96	13	72.35 ± 156.36	effect size	0.065	0.046	0.056
IL1b	Hypercapnic	10	17.60 ± 18.44	10	12.82 ± 10.80	*p*-value	0.266	0.210	0.130
	Normocapnic	10	11.89 ± 12.92	11	33.70 ± 43.93	effect size	0.050	0.073	0.106
IL8	Hypercapnic	16	34.07 ± 27.97	13	24.14 ± 19.38	*p*-value	0.309	0.591	0.383
	Normocapnic	13	41.88 ± 32.57	16	39.42 ± 40.27	effect size	0.021	0.008	0.020

rmANOVA—ANOVA for repeated measures.

**Table 4:** Inflammatory markers from serum in the hypercapnic and normocapnic group.

Marker	Group	Before		After		rmANOVA Analysis			
		N	Mean ± SD	N	Mean ± SD		Group	Time	Group: Time
WBC	Hypercapnic	13	7.44± 3.95	17	11.19± 5.20	*p*-value	0.469	<0.001	0.007
	Normocapnic	11	7.92± 4.09	15	8.78± 4.53	effect size	0.012	0.065	0.027
CRP	Hypercapnic	13	11.88± 25.71	17	11.00± 23.09	*p*-value	0.732	0.938	0.177
	Normocapnic	14	9.06± 8.46	13	9.85± 8.54	effect size	0.003	<0.001	0.001
IL6	Hypercapnic	16	15.52± 18.59	13	47.87± 40.76	*p*-value	0.002	<0.001	0.027
	Normocapnic	13	7.00± 4.64	16	16.99± 15.84	effect size	0.152	0.171	0.055

rmANOVA—ANOVA for repeated measures.

**Table 5:** Univariable linear regression factors for postoperative IL-6.

	Standardized Coefficients Beta	Unstandardized Coefficients B	95%CI for B	*p*-Value
Groups	−0.456	−30.884	−50.679–−11.089	0.003
Smoking status	0.228	15.764	−6.339–37.886	0.157
Thoracotomy/VATS	−0.304	−20.744	−43.069–1.580	0.068
Duration of surgery	0.559	0.301	0.169–0.433	<0.001
Duration of one lung ventilation	0.662	0.356	0.203–0.489	<0.001
Duration of mechanical ventilation	0.590	0.276	0.152–0.400	<0.001
Blood loss	0.281	0.079	−0.009–0.165	0.079

**Table 6:** Exploratory adjusted linear regression models for factors associated with postoperative serum IL-6 levels.

		Standardized Coefficients Beta	Unstandardized Coefficients B	95%CI for B	*p*-Value
	Groups	−0.456	−30.884	−50.679–−11.089	0.003
	Duration of surgery	0.559	0.301	0.169–0.433	<0.001
Model 2	Groups	−0.344	−23.327	−39.608–−7.046	0.006
	One lung ventilation	0.552	0.307	0.173–0.441	<0.001
Model 3	Groups	−0.437	−22.834	−50.679–−11.089	0.010
	Duration of mechanical ventilation	0.512	0.240	0.152–0.400	<0.001

**Table 7:** Intraoperative and postoperative complications by subgroups.

Complications	Hypercapnic Group		Normocapnic Group		*p* *
	PC	VC	PC	VC	
No	1 (10.0%)	3 (30.0%)	6 (60.0%)	3 (30.0%)	
Yes	9 (90.0%)	7 (70.0%)	4 (40.0%)	7 (70.0%)	0.121
Drop in oxygen saturation	7 (70.0%)	6 (60.0%)	3 (30.0%)	5 (50.0%)	0.320
Agitation	1 (10.0%)	1 (10.0%)	0 (0.0%)	1 (10.0%)	0.790

* Chi-square test.

## Data Availability

The original contributions presented in this study are included in the article/Supplementary Material. Further inquiries can be directed to the corresponding author.

## Supplementary Materials

The following supporting information can be downloaded at https://www.mdpi.com/article/10.3390/metabo16080550/s1, Table S1: Inflammatory markers in BAL according to ventilation mode (VC vs. PC) in hypercapnic and normocapnic group; Table S2: Inflammatory markers in serum in the hypercapnic and normocapnic group were divided into VC and PC subgroups.

## References

[1] Lohser J., Slinger P. (2015). Lung Injury After One-Lung Ventilation: A Review of the Pathophysiologic Mechanisms Affecting the Ventilated and the Collapsed Lung. Anesth. Analg..

[2] Amato M.B., Barbas C.S., Medeiros D.M., Magaldi R.B., Schettino G.P., Lorenzi-Filho G., Kairalla R., Deheinzelin D., Munoz C., Oliveira R. (1998). Effect of a protective-ventilation strategy on mortality in the acute respiratory distress syndrome. N. Engl. J. Med..

[3] Kozian A., Schilling T., Fredén F., Maripuu E., Röcken C., Strang C., Hachenberg T., Hedenstierna G. (2008). One-lung ventilation induces hyperperfusion and alveolar damage in the ventilated lung: An experimental study. Br. J. Anaesth..

[4] Schilling T., Kozian A., Senturk M., Huth C., Reinhold A., Hedenstierna G., Hachenberg T. (2011). Effects of volatile and intravenous anesthesia on the alveolar and systemic inflammatory response in thoracic surgical patients. Anesthesiology.

[5] Brower R.G., Matthay M.A., Morris A., Schoenfeld D., Thompson B.T., Wheeler A. (2000). Acute Respiratory Distress Syndrome Network. Ventilation with lower tidal volumes as compared with traditional tidal volumes for acute lung injury and the acute respiratory distress syndrome. N. Engl. J. Med..

[6] Zhang Z., Hu X., Zhang X., Zhu X., Chen L., Zhu L., Hu C., Du B. (2015). China Critical Care Clinical Trials Group (CCCCTG).Lung protective ventilation in patients undergoing major surgery: A systematic review incorporating a Bayesian approach. BMJ Open.

[7] Mosier J.M., Hypes C., Joshi R., Whitmore S., Parthasarathy S., Cairns C.B. (2015). Ventilator Strategies and Rescue Therapies for Management of Acute Respiratory Failure in the Emergency Department. Ann. Emerg. Med..

[8] Laffey J.G., Kavanagh B.P. (1999). Carbon dioxide and the critically ill—Too little of a good thing?. Lancet.

[9] Laffey J.G., Honan D., Hopkins N., Hyvelin J.M., Boylan J.F., McLoughlin P. (2004). Hypercapnic Acidosis Attenuates Endotoxin-Induced Acute Lung Injury. Am. J. Respir. Crit. Care Med..

[10] Gao W., Liu D.D., Li D., Cui G.X. (2015). Effect of Therapeutic Hypercapnia on Inflammatory Responses to One-lung Ventilation in Lobectomy Patients. Anesthesiology.

[11] Sinclair S.E., Kregenow D.A., Lamm W.J.E., Starr I.R., Chi E.Y., Hlastala M.P. (2002). Hypercapnic Acidosis Is Protective in an In Vivo Model of Ventilator-induced Lung Injury. Am. J. Respir. Crit. Care Med..

[12] Dada L.A., Trejo Bittar H.E., Welch L.C., Vagin O., Deiss-Yehiely N., Kelly A.M., Baker M.R., Capri J., Cohn W., Whitelegge J.P. (2015). High CO_2_ Leads to Na, K-ATPase Endocytosis via c-Jun Amino-Terminal Kinase-Induced LMO7b Phosphorylation. Mol. Cell. Biol..

[13] Vadász I., Cummins E.P., Brotherton D.H., Casalino-Matsuda S.M., Dada L.A., Green O., King D.T., Kryvenko V., Shigemura M., Sporn P.H.S. (2025). Sensing Molecular Carbon Dioxide: A Translational Focus for Respiratory Disease. Physiol. Rev..

[14] Csoma B., Vulpi M.R., Dragonieri S., Bentley A., Felton T., Lázár Z., Bikov A. (2022). Hypercapnia in COPD: Causes, Consequences, and Therapy. J. Clin. Med..

[15] Mehrotra M., Jain A. (2023). Single-Lung Ventilation. StatPearls [Internet].

[16] Bae V., Choi S., Lee J., Park J., Lee C.H., Yim J.J., Kim Y., Han S., Yoo C.G. (2014). Clinical outcomes of acute respiratory distress syndromeafter pulmonary resectionin lung cancer patients. Am. J. Resp. Crit. Care Med..

[17] Brower R.G., Lanken P.N., MacIntyre N., Matthay M.A., Morris A., Ancukiewicz M., Schoenfeld D., Thompson B.T. (2004). National Heart, Lung, and Blood Institute ARDS Clinical Trials Network. Higher versus lower positive end-expiratory pressures in patients with the acute respiratory distress syndrome. N. Engl. J. Med..

[18] Ismaiel N., Whynot S., Geldenhuys L., Xu Z., Slutsky A.Z., Chappe V., Henzler D. (2022). Lung-Protective Ventilation Attenuates Mechanical Injury While Hypercapnia Attenuates Biological Injury in a Rat Model of Ventilator-Associated Lung Injury. Front. Physiol..

[19] Ge Y., Yuan L., Jiang X., Wang X., Xu R., Ma W. (2013). Effect of lung protection mechanical ventilation on respiratory function in the elderly undergoing spinal fusion. Zhong Nan DA Xue Xue Bao YI Xue Ban.

[20] Ferrando C., Carramiñana A., Piñeiro P., Mirabella L., Spadaro S., Librero J., Ramasco F., Scaramuzzo G., Cervantes O., Garutti I. (2024). IPROVE-OLV Research Network Group. Individualised, perioperative open-lung ventilation strategy during one-lung ventilation (iPROVE-OLV): A multicentre, randomised, controlled clinical trial. Lancet Respir. Med..

[21] Writing Committee, Steering Committee, PROTHOR Collaborative Group, PROtective VEntilation Network (PROVE Network) for the Clinical Trial Network of the European Society of Anaesthesiology and Intensive Care (2026). Effects of intraoperative higher versus lower positive end-expiratory pressure during one-lung ventilation for thoracic surgery on postoperative pulmonary complications (PROTHOR): A multicentre, international, randomised, controlled, phase 3 trial. Lancet Respir. Med..

[22] Hickling K.G., Walsh J., Henderson S., Jackson R. (1994). Low Mortality Rate in Adult Respiratory Distress Syndrome Using Low- Volume, Pressure-Limited Ventilation with Permissive Hypercapnia: A Prospective Study. Crit. Care Med..

[23] Contreras M., Masterson C., Laffey J.G. (2015). Permissive Hypercapnia. Curr. Opin. Anaesthesiol..

[24] Laffey J.G., Kavanagh B.P. (2000). Biological effects of hypercapnia. Intensive Care Med..

[25] Darioli R., Perret C. (1984). Mechanical Controlled Hypoventilation in Status Asthmaticus. Am. Rev. Respir. Dis..

[26] Shibata K., Cregg N., Engelberts D., Takeuchi A., Fedorko L., Kavanagh B.P. (1998). Hypercapnic Acidosis May Attenuate Acute Lung Injury by Inhibition of Endogenous Xanthine Oxidase. Am. J. Respir. Crit. Care Med..

[27] Jawa R.S., Anillo S., Huntoon K., Baumann H., Kulaylat M. (2011). Interleukin-6 in surgery, trauma, and critical care part II: Clinical implications. J. Intensive Care Med..

[28] Castell J.V., Gomez-Lechon M.J., David M., Andus T., Geiger T., Trullenque R., Fabra R., Heinrich P.C. (1989). Interleukin-6 is the major regulator of acute phase protein synthesis in adult human hepatocytes. FEBS Lett..

[29] Cesta M.C., Zippoli M., Marsiglia C., Gavioli E.M., Mantelli F., Allegretti M., Balk R.A. (2021). The role of interleukin-8 in lung inflammation and injury: Implications for the management of COVID-19 and hyperinflammatory acute respiratory distress syndrome. Front. Pharmacol..

[30] Klava A., Windsor A.C., Farmery S.M., Woodhouse L.F., Reynolds J.V., Ramsden C.W., Boylston A.W., Guillou P.J. (1997). Interleukin 10. A role in the development of postoperative immunosuppression. Arch. Surg..

[31] Wen X.H., Kong H.Y., Zhu S.M., Xu J.H., Huang S.Q., Chen Q.L. (2004). Plasma levels of tumor necrotic factor-alpha and interleukin-6,-8 during orthotopic liver transplantation and their relations to postoperative pulmonary complications. Hepatobiliary Pancreat. Dis. Int..

[32] DelaGala F., Pineiro P., Reyes A., Vara E., Olmedilla L., Cruz P., Garutti I. (2017). Postoperative pulmonary complications, pulmonary and systemic inflammatory responses after lung resection surgery with prolonged one-lung ventilation. Randomized controlled trial comparing intravenous and inhalational anaesthesia. Br. J. Anaesth..

[33] Broccard A.F., Hotchkiss J.R., Vannay C., Markert M., Sauty A., Feihl F., Schaller M.D. (2001). Protective effects of hypercapnic acidosis on ventilator induced lung injury. Am. J. Respir. Crit. Care Med..

[34] Feihl F., Eckert P., Brimioulle S., Jacobs O., Schaller M.D., Mélot C., Naeije R. (2000). Permissive hypercapnia impairs pulmonary gas exchange in the acute respiratory distress syndrome. Am. J. Respir. Crit. Care Med..

[35] Doerr C.H., Gajic O., Berrios J.C., Caples S., Abdel M., Lymp J.F., Hubmayr R.D. (2005). Hypercapnic acidosis impairs plasma membrane wound resealing in ventilator-injured lungs. Am. J. Respir. Crit. Care Med..

[36] O’Toole D., Hassett P., Contreras M., Higgins B.D., McKeown S.T., McAuley D.F., O’Brien T., Laffey J.G. (2009). Hypercapnic acidosis attenuates pulmonary epithelial wound repair by an NF-kappaB dependent mecha nism. Thorax.

[37] Briva A., Vadász I., Lecuona E., Welch L.C., Chen J., Dada L.A., Trejo H.E., Dumasus V., Azzam Z.S., Myrianthefs P.M. (2007). High CO_2_ levels impair alveolar epithelial function independently of pH. PLoS ONE.

[38] Welch L.C., Lecuona E., Briva A., Trejo H.E., Dada L.A., Sznajder J.I. (2010). Extracellular signal-regulated kinase (ERK) participates in the hypercapnia-induced Na, K-ATPase downregulation. FEBS Lett..

[39] Lecuona E., Sun H., Chen J., Trejo H.E., Baker M.A., Sznajder J.I. (2013). Protein kinase A-Ialpha regulates Na, K-ATPase endocytosis in alveolar epithelial cells exposed to high CO2 concentrations. Am. J. Respir. Cell Mol. Biol..

[40] Vadász I., Dada L.A., Briva A., Helenius I.T., Sharabi K., Welch L.C., Kelly A.M., Grzesik B.A., Scott Budinger G.R., Liu L. (2012). Evolutionary conserved role of c-Jun-Nterminal kinase in CO2-induced epithelial dysfunction. PLoS ONE.

[41] Wang N., Gates K.L., Trejo H., Favoreto S., Schleimer R.P., Sznajder J.I., Beitel G.J., Sporn P.H.S. (2010). Elevated CO2 selectively inhibits interleukin-6 and tumor necrosis factor expression and decreases phagocytosis in the macrophage. FASEB J..

[42] Rotta A.T., Gunnarsson B., Furhman B.P., Hernan L.J., Steinhorn D.M. (2001). Comparison of lung protective ventilation strategies in a rabbit model of acute lung injury. Crit. Care Med..

[43] Lang J., Figueroa M., Sanders K.D., Aslan M., Liu Y., Chumley P., Freeman B.A. (2005). Hypercapnia via reduced rate and tidal volume contributed to lipopolysaccharide-induced lung injury. Am. J. Respir. Crit. Care Med..

[44] Gates K.L., Howell H.A., Nair A., Vohwinkel C.U., Welch L.C., Beitel G.J., Hauser A.R., Sznajder J.I., Sporn P.H. (2013). Hypercapnia impairs lung neutrophil function and increases mortality in murine *Pseudomonas* pneumonia. Am. J. Respir. Cell Mol. Biol..

[45] O’Croinin D.F., Hopkins N.P., Moore M.M., Boylan J.F., McLoughlin P., Laffey J.G. (2005). Hypercapnic acidosis does not modulate the severity of bacterial pneumonia-induced lung injury. Crit. Care Med..

[46] Casalino-Matsuda S.M., Chen F., Gonzalez-Gonzalez F.J., Nair A., Dib S., Yemelyanov A., Gates K.L., Scott Budinger G.R., Beitel G.J., Sporn P.H.S. (2020). Hypercapnia Suppresses Macrophage Antiviral Activity and Increases Mortality of Influenza A Infection via Akt1. J. Immunol..

[47] Cummins E.P., Oliver K.M., Lenihan C.R., Fitzpatrick S.F., Bruning U., Scholz C.C., Slattery C., O Leonard M., McLoughlin P., Taylor C.T. (2010). NF-B links CO2 sensing to innate immunity and in ammation in mammalian cells. J. Immunol..

[48] Oliver K.M., Lenihan C.R., Bruning U., Cheong A., Laffey J.G., McLoughlin P., Taylor C.T., Cummins E.P. (2012). Hypercapnia induces cleavage and nuclear localization of RelB protein, giving insight into CO_2_ sensing and signaling. J. Biol. Chem..

[49] Silvestre C., Vyas H. (2015). Is permissive hypercapnia helpful or harmful?. Paediatr. Child Health.

[50] Bellocq A., Suberville S., Philippe C., Bertrand F., Perez J., Fouqueray B., Cherqui G., Baud L. (1998). Low environmental pH is responsible for the induction of nitric-oxide synthase in macrophages. J. Biol. Chem..

[51] Laffey J.G., O’Croinin D., McLoughlin P., Kavanagh B.P. (2004). Permissive hypercapnia—role in protective lung ventilatory strategies. Intensive Care Med..

[52] Yim A., Wan S., Lee T.W., Arifi A.A. (2000). VATS lobectomy reduces cytokine reponses compared with conventional surgery. Ann. Thorac. Surg..

[53] Ren H., Shang Z., Qi Y., Huang K. (2026). Ventilator-Induced Lung Injury: Mechanotransduction and Potential Therapeutic Targets. MedComm.

[54] Ismaiel M., Henzler D. (2010). Effects of Hypercapnia and Hypercapnic Acidosis on Attenuation of Ventilator-Associated Lung Injury. Minerva Anestesiol..

[55] Gigliotti S., Guerriero G., Mazza G., Garofalo E., Pavia G., Amaddeo A., Rizzuto A., Marascio N., Matera G. (2026). Perioperative Blood Biomarkers of Infectious and Non-Infectious Postoperative Pulmonary Complications: A Narrative Review. J. Clin. Med..

