# Supplementary material for: Effects of Permissive Hypercapnia During Lung Surgery on Inflammatory Biomarkers in Serum and Bronchoalveolar Lavage Fluid: A Randomized Controlled Trial
Source: Metabolites. 2026 Aug 4;16(8):550. doi: 10.3390/metabo16080550 (PMC13515437; doi:10.3390/metabo16080550)
Supplement: Supplementary file 1 [file metabolites-16-00550-s001.zip › metabolites-4455998-supplementary.pdf]

Supplementary Table S1. Inflammatory markers in BAL according to ventilation mode (VC vs. PC) in hypercapnic and normocapnic group.

Inflammatory markers	Hypercapnic group		Normocapnic group		p*
	PC	VC	PC	VC	
IL-6 before(pg/ml)	1.47±1.12	2.81±2.74	0.53±0.37	1.56±1.91	0.174
IL-6 after(pg/ml)	1.35±1.51	3.15±1.90	8.89±11.49	1.41±1.45	0.148
TNF- α after(pg/ml)	9.29±5.26	22.08±37.73	17.84±15.93	14.90±18.22	0.865
TNF-α after(pg/ml)	13.39±12.76	16.21±14.70	89.51±176.42	13.79±12.49	0.396
IL-1β before(pg/ml)	13.87±15.01	21.31±22.48	7.03±5.32	16.74±16.97	0.449
IL-1β after(pg/ml)	14.58±17.73	11.64±4.13	42.82±48.98	9.36±5.43	0.335
IL-8 before(pg/ml)	20.27±11.87	53.74±31.60	46.27±34.68	21.73±17.97	0.115
IL-8 after(pg/ml)	15.59±5.46[a]	31.46±24.30	59.50±43.97[b]	13.59±9.59	0.032

*Kruskal-Wallis test; [a] Mann-Whitney test (PC in hypercapnic group vs. PC in normocapnic group); [b] PC vs. VC in normocapnic group

Supplementary Table S2. Inflammatory markers in serum in the hypercapnic and normocapnic group were divided into VC and PC subgroups.

Marker	**Measurement**	Hypercapnic group		Normocapnic group		p*
		PC	VC	PC	VC	
WBC (×10⁹/L)	before	7.07 ± 2.74	7.81 ± 5.00	7.50 ±3.97	8.33 ± 4.38	0.916
	after	11.71 ± 4.21	10.66 ± 6.22	9.00 ± 3.47	8.56 ± 5.58	0.566
CRP (mg/L)	before	4.18 ± 3.11	19.58 ± 35.42	9.76 ± 10.05	8.37 ± 7.00	0.197
	after	4.29 ± 3.23	17.71 ± 31.86	9.59 ± 9.54	10.11 ± 7.93	0.240
IL-6 (pg/ml)	before	21.47 ± 23.28	9.56 ± 10.44	6.78 ± 3.53	7.22 ± 5.74	0.554
	after	65.40 ± 48.24	30.34 ± 22.29	22.11 ± 20.79[a]	11.85 ± 6.26[a]	0.006

*Kruskal-Wallis test; [a] Mann-Whitney test (p<0.05 vs. PC in hypercapnic group)
